# Effect of Computer Navigation on Accuracy and Reliability of Limb Alignment Correction following Open-Wedge High Tibial Osteotomy: A Meta-Analysis

**DOI:** 10.1155/2017/3803457

**Published:** 2017-10-09

**Authors:** Seung-Beom Han, Hyun Jung Kim, Dae-Hee Lee

**Affiliations:** ^1^Department of Orthopaedic Surgery, Korea University Anam Hospital, Korea University College of Medicine, Seoul, Republic of Korea; ^2^Department of Preventive Medicine, Korea University College of Medicine, Seoul, Republic of Korea; ^3^Department of Orthopaedic Surgery, Samsung Medical Center, Sungkyunkwan University School of Medicine, Seoul, Republic of Korea

## Abstract

**Background:**

It is unclear whether computer navigation can improve the accuracy and reliability of targeted lower limb alignment correction following open-wedge high tibial osteotomy (HTO). This meta-analysis was designed to compare the accuracy and reliability of limb alignment correction between computer navigated and conventional open-wedge HTOs.

**Methods:**

Studies that compared postoperative coronal alignment, including mechanical axis (MA) and weight bearing line (WBL) ratio, outliers of alignment correction, and change in tibial posterior slope, following open-wedge HTO performed using computer navigated and conventional methods were included.

**Results:**

Ten studies were included in the meta-analysis. The MA (0.93°; 95% confidence interval [CI]: 0.45–1.41°; *P* < 0.001) and WBL ratio (1.5%; 95% CI: 0.03–2.98%; *P* = 0.048) were significantly greater for computer navigated HTO than for conventional HTO. Outliers of alignment correction after surgery were significantly lower in patients who underwent computer navigated HTO than in those who underwent conventional HTO (odds ratio: 0.25; 95% CI: 0.08–0.79; *P* = 0.02). Changes in posterior tibial slope from before to after surgery, however, were similar for the two approaches.

**Conclusion:**

Computer navigated HTO resulted in slightly more valgus postoperative alignment and effectively reduced outliers of alignment correction but had no effect on change in posterior tibial slope when compared with conventional HTO.

## 1. Introduction

Open-wedge high tibial osteotomy (HTO) is easier to perform and is more adjustable for alignment correction than closed-wedge HTO; also it has the advantage of avoiding the complications associated with closed-wedge HTO, such as peroneal nerve palsy [1, 2]. Although accurate alignment correction is a key factor for achieving successful open-wedge HTO [3–5], preoperatively planned optimal correction of alignment is difficult to determine during surgery [6], because the lower limb is covered with sterile drapes and wrapped by a tourniquet [7]. Many procedures have been utilized to determine intraoperative alignment correction, such as cable, grids, and a jig-based system under fluoroscopy, but none has proven satisfactory to date.

Computer navigation has been shown to be effective for accurate restoration of neutral alignment in patients undergoing total knee arthroplasty (TKA) [8], suggesting that computer navigation may be utilized to determine the intraoperative adequacy of alignment correction during open-wedge HTO [9]. Unlike TKA, in which alignment is determined by direct osseous contact, the indirect skin contact registration process for bony landmarks in open-wedge HTO may lead to different outcomes. In addition, previous studies comparing computer navigated and conventional open-wedge HTOs have shown conflicting results. This meta-analysis was therefore designed to compare the accuracy and reliability of limb alignment correction between computer navigated and conventional open-wedge HTOs. It was hypothesized that the two approaches would be similarly accurate and reliable.

## 2. Methods

### 2.1. Data and Literature Sources

The study design was based on Cochrane Review Methods. Multiple comprehensive databases, including MEDLINE (1 January 1976 to 31 May 2015), EMBASE (1 January 1985 to 31 May 2015), the Cochrane Library (1 January 1987 to 31 May 2015), and KoreaMed (1 June 1958 to 31 May 2015), were searched for studies that compared graft extrusion on postoperative MRI in patients who underwent medial and lateral MATs. There were no restrictions on language or year of publication. Search terms used in the title, abstract, MeSH, and keywords fields included “Osteotomy” [tiab] OR “Tibial” [tiab] OR “High” [tiab] OR “Navigation” [tiab], OR “Open” [tiab], AND “Osteotomy” [MeSH] OR “Computer-assisted” [tiab]. After the initial electronic search, relevant articles and their bibliographies were searched manually. Articles identified were assessed individually for inclusion.

### 2.2. Study Selection

Study inclusion was decided independently by two reviewers, based on the predefined selection criteria. Titles and abstracts were read; if suitability could not be determined, the full article was evaluated. Studies were included in the meta-analysis if they compared alignment correction in patients who underwent open-wedge HTO with and without computer navigation; they simultaneously reported direct comparisons of computer navigation and conventional HTOs; and their primary outcomes included comparisons of coronal alignment after surgery, including mechanical axis (MA), weight bearing line (WBL) ratio, outliers of coronal alignment, and/or changes in posterior slope from before to after surgery. MA was defined as the angle subtended by a line drawn from the center of the femoral head to the center of the tibial spines and a line drawn from the center of the tibial spines to the center of the talus, and WBL was defined as a line drawn from the center of the femoral head to the center of the superior articular surface of the talus. WBL ratio was calculated as the tibial intersection of the WBL/tibial width, with the medial tibial edge defined as 0% and the lateral tibial edge as 100%. Outliers of coronal alignment were defined as deviations from the acceptable range or tolerance of the targeted alignment correction. The angle of the posterior tibial slope on knee lateral radiographs was defined as the angle between the proximal plateau and a line drawn perpendicular to the tibial shaft axis. Studies were also included in the meta-analysis if they fully reported the number of knees in each group (computer navigated versus conventional groups), the means and standard deviations of parameters of alignment correction and posterior slope change, and the numbers that deviated from the acceptable range of targeted alignment correction in each group and if they used adequate statistical methods to compare alignment parameters, outliers of alignment correction, and posterior slope change in the two groups.

### 2.3. Data Extraction

Two reviewers independently recorded data from each study using a predefined data extraction form. Any disagreement unresolved by discussion was reviewed by a third author. Variables recorded included (1) means and standard deviations of postoperative MA or WBL ratio and numbers of outliers of coronal alignment following computer navigated and conventional HTO, (2) complications during or following open-wedge HTO such as lateral tibial cortical fractures and delayed union or nonunion, (3) the sample size of each group, and (4) the type of computer navigation system. If these variables were not mentioned in the articles, the study's authors were contacted by email to request these data.

### 2.4. Assessment of Methodological Quality

Two reviewers independently assessed the methodological quality of each study using the Newcastle-Ottawa Scale, as recommended by the Cochrane Non-Randomized Studies Methods Working Group. In this study, the Newcastle-Ottawa Scale's star system, which awards stars depending on the level of bias, was adjusted to a scale that included only low (one star), high, and unclear bias. Each study was judged on three criteria: the selection of the study groups, the comparability of the groups, and the ascertainment of either the exposure or outcome of interest for case-control or cohort studies. Any unresolved disagreements between reviewers were resolved by consensus or by consultation with a third investigator.

### 2.5. Statistical Analysis

The main outcomes of the meta-analysis were the mean differences in MA and WBL ratio, change in posterior slope from before to after surgery, and the proportion of outliers of coronal alignment between groups of knees that underwent computer navigated and conventional HTO. Binary outcomes were reported as odds ratios (ORs) and 95% confidence intervals (CIs), whereas continuous outcomes comparing medial and lateral MATs were reported as mean differences and 95% CIs. Heterogeneity was determined by estimating the proportion of between-study inconsistencies due to actual differences between studies rather than differences due to random error or chance, using the *I*^2^ statistic, with values of 25%, 50%, and 75% considered low, moderate, and high, respectively. All statistical analyses were performed using RevMan version 5.2 and Stata/MP 13.0.

## 3. Results

### 3.1. Identification of Studies

**Figure 1:**
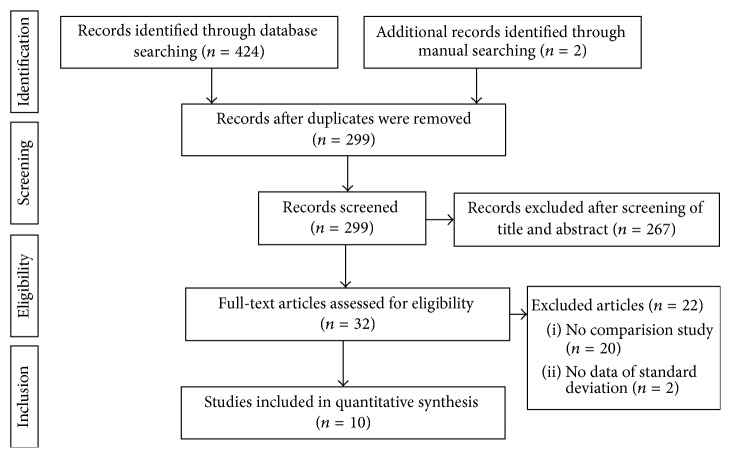
PRISMA (Preferred Reporting Items for Systematic Reviews and Meta-analyses) flow diagram of the identification and selection of the studies included in this meta-analysis.

Figure 1 shows the details of study identification, inclusion, and exclusion. Electronic searches yielded 179 studies in PubMed (MEDLINE), 235 in EMBASE, and 10 in the Cochrane Library. Two additional publications were identified through manual searching. After removing 127 duplications, 299 studies remained; of these, 267 were excluded based on reading of the abstracts and full-text articles. An additional 20 studies were excluded, since they included subjects who underwent computer navigated or conventional HTO, not both, and two were excluded because they did not report standard deviations of alignment. After applying these criteria, 10 studies were finally included in this meta-analysis.

### 3.2. Study Characteristics and Quality Assessment

The 10 included studies involved 275 knees that underwent computer navigated open-wedge HTO and 251 knees that underwent conventional open-wedge HTO. The 10 studies retrospectively compared combinations of four parameters: absolute coronal alignment, including MA and/or WBL ratio, proportion of outliers of alignment correction after open-wedge HTO, and change in tibial posterior slope from before to after surgery. Three studies measured three parameters, five compared two parameters, and two compared one parameter each. Four of the 10 included studies reported complications, which included lateral cortex fracture, delayed union, and a broken screw on the plate (Table 1). All ten studies included in this meta-analysis had a low risk of selection bias and compared demographic data between knees that underwent computer navigated and conventional open-wedge HTO; none, however, assessed possible confounding factors. Sufficient follow-up duration was defined as the time from surgery to taking radiographs, with shorter time intervals associated with a higher risk of bias, because early follow-up radiographs could not measure actual alignment correction due to remaining flexion contracture. If postoperative radiographs were taken within three months after surgery, that study was considered as having a high risk of bias.  Table 2 summarizes the risk of bias for the 10 studies included in this meta-analysis.

### 3.3. Coronal Alignment

**Figure 2:**
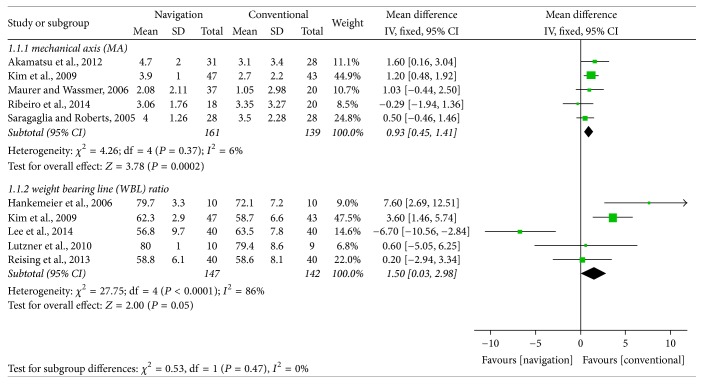
Forest plot showing the mean differences in coronal alignments, including mechanical axis and weight bearing line ratio, between computer navigated and conventional high tibial osteotomies.

Of the 10 studies, 9 compared postoperative coronal alignment correction between computer navigated and conventional HTO, as determined by measuring the MA or WBL ratio. These nine studies included 261 subjects who underwent computer navigated HTO and 238 who underwent conventional HTO. Pooled data showed that MA was 0.93° greater in computer navigated than in conventional HTO (95% CI: 0.45°–1.41°; *P* < 0.001; *I*^2^ = 6%) and that WBL ratio from the medial edge of the tibial plateau was 1.5% greater in computer navigated than in conventional HTO (95% CI: 0.03%–2.98%; *P* = 0.048; *I*^2^ = 86%, Figure 2). These findings indicated that computer navigated HTO resulted in slightly more valgus coronal alignment than conventional HTO.

### 3.4. Outliers of Alignment

**Figure 3:**
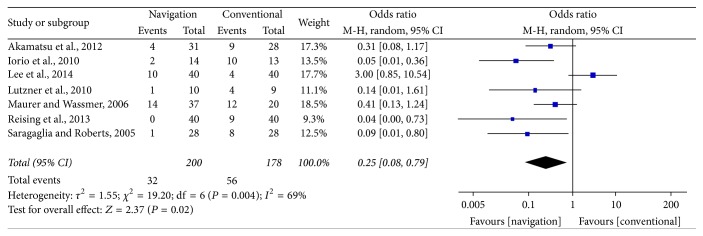
Forest plot showing the proportion of outliers of alignment (>±3°) between computer navigated and conventional high tibial osteotomies.

Of the 10 studies, seven reported the proportion of knees deviated from correction target (MA, 2° to 8°, or WBL ratio, 50% to 70%). The pooled results showed that the proportion of postoperative outliers of alignment correction was significantly lower in patients who underwent computer navigated than conventional HTO (32/200 [16.0%] versus 56/178 [31.5%]; OR: 0.25; 95% CI: 0.08–0.79; *P* = 0.02; *I*^2^ = 69%, Figure 3).

### 3.5. Change of Posterior Tibial Slope

**Figure 4:**
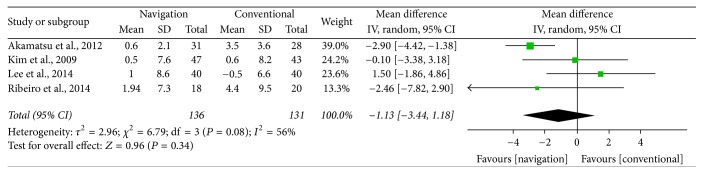
Forest plot showing the mean differences in posterior tibial slope between computer navigated and conventional high tibial osteotomies.

Four studies compared changes from baseline in posterior tibial slope following computer navigated and conventional HTO. These studies included 136 subjects who underwent computer navigated HTO and 131 who underwent conventional HTO. The pooled results showed that the change in posterior tibial slope was 1.13° smaller with computer navigated than with conventional HTO, but this difference was not statistically significant (95% CI: −3.44°–1.18°; *P* = 0.34; *I*^2^ = 56%, Figure 4).

## 4. Discussion

The most important findings of this meta-analysis were that computer navigated HTO tended to result in more valgus alignment and reduced the percentage of outliers of alignment correction after surgery than did conventional HTO.

Although computer navigated HTO resulted in slightly more valgus alignment correction, it is unclear whether this more valgus tendency was associated with better clinical outcomes. Nine of the 10 studies included in this meta-analysis used the same computer navigation system (OrthoPilot), with a targeted correction of alignment of 30–40% of the lateral tibial plateau from the center of the knee [19]. In contrast, conventional preoperative planning targeting the amount of correction aimed for the mechanical axis to pass 62.5% laterally from the medial tibial plateau margin [20]. If the medial and lateral tibia plateaus are symmetrical, the target point of the computer navigation system would be 65–70% from the medial tibial plateau margin or 2.5–7.5% more lateral than the target point of preoperative planning [15]. This difference in target points may account in part for the slightly more valgus postoperative alignment in computer navigated than conventional open-wedge HTO.

Another possible reason that computer navigated HTO resulted in a slightly more valgus alignment may be due to differences in weight bearing conditions [21]. Computer navigated HTO measures alignment with the patient in the supine position, without weight bearing. Following osteotomy during open-wedge HTO, the tension in the soft tissue envelope is altered by both the osteotomy and the unavoidable complete or partial release of the superficial medial collateral ligament [22]. After surgery, the soft tissue may therefore be vulnerable to medial laxity of the knee joint when bearing the patient's weight, resulting in a slight valgus alignment in standing whole leg radiographs compared with intraoperative corrected alignment [23].

Although several studies have addressed the effect of the navigation system on the accuracy of alignment correction in HTO, most of these studies compared mean postoperative MA and/or WBL ratio of the lower limb. The accuracy and reliability of a monitoring system, such as surgical computer navigation in the operating room, may be more accurately determined by the number of outliers from the acceptable tolerance of the target point than by comparison of mean values [16]. By pooling the results of several studies, this meta-analysis showed that the percent of alignment correction outliers was significantly lower with computer navigated than with conventional HTO. This finding may be explained by the vulnerability of radiographic alignment to knee position. Errors in alignment correction may be due to both inaccurate preoperative planning and inadequate intraoperative quantitative assessment of the amount of correction, both of which are assessed radiographically or by intraoperative fluoroscopy, which are dependent on correct positioning. Measurement errors due to knee flexion and axial rotation can also occur at the moment a radiograph is taken. Coronal alignment of the knee could be affected by these flexion contractures and/or malrotations [24, 25].

Posterior tibial slope has been reported to increase inadvertently after open-wedge HTO due to the distinguishing anatomical features of the cross-sectional shape of the proximal tibia [26–28]. Four of the studies included in this meta-analysis compared the change in slope from before to after surgery in patients who underwent computer navigated and conventional HTO, finding that posterior slope after surgery was slightly greater using both methods. The meta-analysis, however, found that changes in posterior slope were similar in the two groups. Posterior tibial slope was not monitored in all studies of computer navigation systems included in this meta-analysis. Change in tibial slope following open-wedge HTO may be influenced by several factors, including the completeness of posterior corticotomy, the release of posterior soft tissue, the location of the plate and the lateral hinge, and the ratio of anterior to posterior gaps at the osteotomy site [29, 30]. Advanced versions of computer navigation systems are needed which include protocols that address the inability of current systems to control posterior slope.

This study had several limitations. All of the studies included in this meta-analysis were observational comparison studies. Therefore, there was some inherent heterogeneity due to uncontrolled bias. In addition, there were some differences among studies in surgical techniques (e.g., partial release or complete resection of the medial collateral ligament), acceptable range of alignment correction from the target point, and tool used to measure radiographic parameters (e.g., manual ruler on conventional radiographs or calibrated program on digital radiographs). These factors may explain, at least in part, some of the heterogeneities in the results of this meta-analysis. Another limitation was that the same computer navigation system was used in 9 of the 10 included studies, which may have led to selection bias.

In conclusion, compared with conventional open-wedge HTO, computer navigated open-wedge HTO resulted in slightly more valgus coronal alignment and more reliable lower limb alignment correction within the target range but had no effect on change of posterior tibial slope.

## Figures and Tables

**Table 1:** Characteristics of the studies included in the meta-analysis.

Authors	Year	Study type	Sample size		Navigation system	Measured parameters	Target alignment	Lateral cortex fracture	Other complications
			Navigation	Conventional					
Akamatsu et al. [10]	2012	RCS	31	28	OrthoPilot	MA, CAO, TPS	4° valgus	4 (conventional) 5 (navigation)	1 delayed union (conventional)
Hankemeier et al. [1]	2006	RCS	10	10	Medivision	WBLR	WBLR 80%	No	NR
Iorio et al. [11]	2010	RCS	14	13	OrthoPilot	CAO	2°–6° valgus	NR	2 broken screw (conventional)
Kim et al. [12]	2009	RCS	47	43	OrthoPilot	MA, WBLR, TPS	3°–5° valgus WBLR 62%	2 (conventional) 3 (navigation)	2 delayed union (conventional), 2 delayed union (navigation), 1 varus collapse (navigation)
Lee et al. [13]	2014	RCS	40	40	OrthoPilot	WBLR, CAO, TPS	WBLR 62% (50%–70%)	NR	NR
Lutzner et al. [14]	2010	RCS	10	9	OrthoPilot	WBLR, CAO	WBLR 80%	NR	NR
Maurer and Wassmer [15]	2006	RCS	37	20	OrthoPilot	MA, CAO	3° valgus (2°–5° valgus)	NR	NR
Reising et al. [16]	2013	RCS	40	40	OrthoPilot	WBLR, CAO	WBLR 62% (50%–70%)	NR	NR
Ribeiro et al. [17]	2014	RCS	18	20	OrthoPilot	MA, TPS	3°–6° valgus	NR	NR
Saragaglia and Roberts [18]	2005	RCS	28	28	OrthoPilot	MA, CAO	3°–6° valgus (HKA 183°–186°)	NR	NR

RCS, retrospective comparison study; MA, mechanical axis; CAO, coronal alignment outlier; WBLR, weight bearing line ratio; TPS, tibial posterior slope; NR, not reported.

**Table 2:** Risk of bias summary: review authors' judgments about each risk of bias item for each included study.

Authors	Representativeness of the cases	Selection of control	Ascertainment of exposure	Interest outcome not present at start of study	Comparability of cohorts	Control for any additional factor	Assessment of outcome	Sufficient follow-up	Adequacy of follow-up
Akamatsu et al. [10]	−	−	−	−	+	+	+	+	−
Hankemeier et al. [1]	−	−	−	−	+	+	−	−	−
Iorio et al. [11]	−	−	−	−	+	+	+	+	−
Kim et al. [12]	−	−	−	−	+	+	+	+	−
Lee et al. [13]	−	−	−	−	+	+	+	+	−
Lutzner et al. [14]	−	−	−	−	−	+	+	+	−
Maurer and Wassmer [15]	−	−	−	−	−	+	+	−	−
Reising et al. [16]	−	−	−	−	−	+	+	−	−
Ribeiro et al. [17]	−	−	−	−	−	+	+	−	−
Saragaglia and Roberts [18]	−	−	−	−	−	+	+	−	−

+, low risk of bias; −, high risk of bias; ?, unclear risk of bias.
